# The association between age at breast cancer diagnosis and prevalence of pathogenic variants

**DOI:** 10.1007/s10549-023-06946-8

**Published:** 2023-04-21

**Authors:** Mary B. Daly, Eric Rosenthal, Shelly Cummings, Ryan Bernhisel, John Kidd, Elisha Hughes, Alexander Gutin, Stephanie Meek, Thomas P. Slavin, Allison W. Kurian

**Affiliations:** 1 Fox Chase Cancer Center, 333 Cottman Avenue, Philadelphia, PA 19111-2497 USA; 2 Myriad Genetics, Inc., Salt Lake City, UT USA; 3 Stanford University School of Medicine, Stanford, CA USA

**Keywords:** Breast cancer, Genetic testing, Cancer risk

## Abstract

**Purpose:**

Young age at breast cancer (BC) diagnosis and family history of BC are strongly associated with high prevalence of pathogenic variants (PVs) in *BRCA1* and *BRCA2* genes. There is limited evidence for such associations with moderate/high penetrance BC-risk genes such as *ATM*, *CHEK2*, and *PALB2*.

**Methods:**

We analyzed multi-gene panel testing results (09/2013–12/2019) for women unaffected by any cancer (N = 371,594) and those affected with BC (N = 130,151) ascertained for suspicion of hereditary breast and/or ovarian cancer. Multivariable logistic regression was used to test association between PV status and age at BC diagnosis (≤ 45 vs. > 45 years) or family history of BC after controlling for personal/family non-BC histories and self-reported ancestry.

**Results:**

An association between young age (≤ 45 years) at diagnosis and presence of PVs was strong for *BRCA1* (OR 3.95, 95% CI 3.64–4.29) and moderate for *BRCA2* (OR 1.98, 95% CI 1.84–2.14). Modest associations were observed between PVs and young age at diagnosis for *ATM* (OR 1.22, 95% CI 1.08–1.37) and *CHEK2* (OR 1.34, 95% CI 1.21–1.47) genes, but not for *PALB2* (OR 1.12, 95% CI 0.98–1.27). For women with BC, earliest age of familial BC diagnosis followed a similar pattern. For unaffected women, earliest age of family cancer diagnosis was significantly associated with PV status only for *BRCA1* (OR 2.34, 95% CI 2.13–2.56) and *BRCA2* (OR 1.25, 95% CI 1.16–1.35).

**Conclusions:**

Young age at BC diagnosis is not a strong risk factor for carrying PVs in BC-associated genes *ATM, CHEK2,* or *PALB2*.

**Supplementary Information:**

The online version contains supplementary material available at 10.1007/s10549-023-06946-8.

## Introduction

Since the identification of the hereditary breast and ovarian cancer genes *BRCA1* and *BRCA2* (*BRCA1/2*) in 1994–5 [1, 2], the concept of hereditary cancer syndromes has been widely accepted by the medical community. Commercial testing for *BRCA1/2* became available in the mid-1990s and its uptake has continued to increase over the past 25 years. This has allowed investigators to describe the phenotype of *BRCA1*/*2* in terms of penetrance, age at diagnosis, strength of family history, histologic subtypes, and association with other cancers [3]. These features have formed the basis of developing criteria for guiding the decision regarding to whom and when to offer genetic testing [4]. By entering the mainstream of clinical practice, genetic testing for *BRCA1/2* has allowed individuals with cancer or an increased risk of cancer to choose personalized prevention, surveillance or treatment options which are appropriate for their level of risk and which are intended to reduce morbidity and mortality.

As data on the prevalence of *BRCA1/2* mutations matured, it became evident that pathogenic variants (PVs) in these genes accounted for less than two-thirds of hereditary breast cancer, leading to a search for additional breast cancer predisposition genes [5]. In 2013, several commercial testing laboratories began to offer multi-gene panels that included an expanded array of genes associated with increased risk for breast and ovarian cancer, as well as other cancers [6]. As the use of multi-gene panels gained wider acceptance, several studies revealed that these panels identified PVs in known breast cancer-risk genes in approximately 8%–15% of women tested. While PVs in *BRCA1/2* accounted for 50%–60% of these variants, an additional 40%–50% were found in other genes, most commonly *ATM, CHEK2,* and *PALB2* [3, 5, 7–15].

National Comprehensive Cancer Network (NCCN) evaluation and testing criteria for hereditary breast and ovarian cancer have relied primarily on the features of *BRCA1/2-*related cancers with a strong emphasis on age of diagnosis and strength of the cancer family history. These criteria continue to evolve with the more recent inclusion of the subtype of the cancer, and potential eligibility for PARP inhibitor therapy [4]. Conversely, the American Society of Breast Surgeons guideline is less prescriptive, with a recommendation for genetic testing in all women with breast cancer, regardless of age at diagnosis or family history [16]. A more precise estimate of the contribution of age at diagnosis of breast cancer to risk of identifying a mutation in a breast cancer gene will help to inform this dialogue.

Efforts are underway to better characterize the patterns associated with PVs in non-*BRCA1/2* breast cancer-predisposition genes in order to describe gene-specific risk factors for carrying a PV and to inform testing guidelines across all women with breast cancer or a familial cancer risk [3]. In addition to family history, one risk factor included in all current testing criteria is early age at onset of breast cancer. While *BRCA1*/*2*-related breast cancers are known to occur at earlier ages than sporadic breast cancers, preliminary data suggest that age at diagnosis observed in patients carrying PVs in other breast cancer risk-associated genes may be more similar to age at diagnosis of sporadic breast cancers [17–19].

This study evaluated the effect of age at breast cancer diagnosis and cancer family history on the risk of carrying a PV in breast cancer susceptibility genes routinely included in breast cancer predisposition multi-gene panels, *ATM, BRCA1, BRCA2, CHEK2,* and *PALB2.*

## Materials and methods

### Cohort

Analysis was performed in a clinical cohort of women ascertained for clinician-suspected hereditary breast/ovarian cancer who received hereditary pan-cancer multi-gene panel testing between September 2013 and December 2019 at a single laboratory. Patients provided informed consent for testing and all de-identified clinical information was obtained from test requisition forms, including patient self-reported ancestry, personal and family cancer history [including type(s) and age(s) at diagnosis]. This analysis did not meet the requirements for studies involving human subjects and was therefore not subject to oversight by an institutional review board.

Two subsets of women were selected for analysis: women with a personal diagnosis of breast cancer, and women unaffected with cancer of any type. Women were excluded if (1) they resided in states with laws preventing the use of de-identified genetic data (Alaska, Colorado, Florida, Michigan, Nebraska, New York, New Hampshire, Oregon, Oklahoma, and South Dakota), (2) carried more than one PV in any tested gene (including homozygous *CHEK2*), (3) had a history of prior genetic testing for founder or familial mutations, (4) had a history of breast cancer but were missing age of diagnosis or (5) had a relative with a diagnosis of breast cancer under the age of 20, based on features suggestive of Li-Fraumeni Syndrome [4]. The final cohort included 501,745 women: 130,151 (25.9%) affected with breast cancer and 371,594 (74.1%) unaffected women.

### Genetic testing

Testing was performed at a Clinical Laboratory Improvement Amendments (CLIA) and College of American Pathology-approved (CAP) laboratory (Myriad Genetic Laboratories, Inc., Salt Lake City, Utah). The initial multi-gene panel test included 25 genes (*APC*, *ATM*, *BARD1*, *BMPR1A*, *BRCA1*, *BRCA2*, *BRIP1*, *CDH1*, *CDK4*, *CHEK2*, *MLH2*, *MSH2*, *MSH6*, *MUTYH*, *NBN*, *P14ARF*, *P16*, *PALB2*, *PMS2*, *PTEN*, *RAD51C*, *RAD51D*, *SMAD4*, *STK11*, and *TP53*). Subsequent additions to the panel test in 2016 and 2019 included *GREM1*, *HOXB13*, *POLD1*, *POLE, AXIN2*, *GALNT12*, *MSH3*, *NTHL1*, *RNF43*, and *RPS20*.

Variant classification was performed using American College of Medical Genetics and Genomics recommendations [20]. Supporting linkage, biochemical, clinical, functional, and statistical data was used to classify variants [21, 22]. Variants that received a classification of Deleterious or Suspected Deleterious were considered PVs. Variants of uncertain significance (VUS) were not included in this analysis, including *CHEK2* c.470C > T (p.Ile157Thr) and c.1283C > T (p.Ser428Phe) [23].

### Statistical methods

Among women affected by breast cancer, multivariable logistic regression was used to evaluate how age at breast cancer diagnosis and family history of female breast cancer affected the likelihood of detecting a PV after controlling for personal and family cancer history (first- and second-degree relatives), and self-reported ancestry. A separate analysis was performed for each gene (*ATM, BRCA1, BRCA2, CHEK2,* and *PALB2)*. PV status was coded as the dependent variable. The multivariable logistic regression models were adjusted using the following independent variables: age of breast cancer diagnosis (≤ 45 years vs > 45 years); personal history of ovarian or pancreatic cancer (coded as binary, affected/unaffected, variables); family history (coded as binary, presence or absence of a first- or second-degree relative) of female breast, male breast, ovarian, pancreatic, and prostate cancer; as well as self-reported ancestry. Self-reported ancestries were coded as quantitative variables representing fractions of reported ancestries as described previously [24].

Additional analyses were performed in women with breast cancer in order to evaluate the effect of earliest age of diagnosis in affected female relatives. These analyses were conducted as above but with binary family history of female breast cancer replaced with four-level family history of female breast cancer (none, earliest age of breast cancer diagnosis ≤ 45 years, earliest age of breast cancer diagnosis > 45 years, age of breast cancer diagnosis unknown).

Among women who were unaffected by cancer of any type, we tested associations of PV status with a presence or absence of breast cancer family history, and with earliest age of diagnosis in affected female relatives using models as described above but excluding the variable for age of personal breast cancer diagnosis.

Confidence intervals (CI) were calculated using Wald statistics. Similar methods have been described previously [24, 25]. All p-values were based on likelihood ratio chi-square test statistics and reported as two-sided. All analyses were performed using R software (R Foundation for Statistical Computing, Vienna, Austria; Version 3.6.1).

## Results

### Cohort characteristics

Demographic and clinical characteristics of the study sample are shown in Table 1**.** The median age at testing was higher for women with a personal history of breast cancer (53 years) compared to unaffected women (41 years). The median age at diagnosis of affected women was 48 years, and over one third (N = 49,218, 37.8%) were diagnosed ≤ 45 years of age. A total of 16,719 (12.8%) affected women reported a second breast cancer. The prevalence of a second breast cancer was similar for those diagnosed ≤ 45 years (12.0%) and those diagnosed > 45 years (13.4%). The percentage of women who met NCCN testing criteria (version 3.2019) was 84% or greater in both subgroups. Self-reported ancestry distributions were similar between women with breast cancer and unaffected women (Table 1).

**Table 1:** Demographics

Variable	Characteristic	Diagnosed ≤ 45 (N = 49,218)	Diagnosed > 45 (N = 80,933)	All Affected with Breast Cancer (N = 130,151)	Unaffected (N = 371,594)
Age at testing	Mean (SD)	45.2 (9.87)	58.7 (8.92)	53.6 (11.38)	41.9 (11.28)
	Median	43	58	53	41
	Q1, Q3	39, 50	51, 64	45, 61	33, 50
	Min, Max	20, ≥ 90	39, ≥ 90	20, ≥ 90	20, ≥ 90
Age at breast cancer Dx	Mean (SD)	39.4 (4.95)	55.9 (8.15)	49.7 (10.72)	–
	Median	41	54	48	–
	Q1, Q3	37, 43	49, 61	42, 57	–
	Min, Max	20, 45	46, ≥ 90	20, ≥ 90	–
Personal history of breast cancer	Breast (1)	43,334 (88.0%)	70,098 (86.6%)	113,432 (87.2%)	–
	Breast (2 +)	5884 (12.0%)	10,835 (13.4%)	16,719 (12.8%)	–
	All affected	49,218 (37.8%)	80,933 (62.2%)	130,151 (100%)	–
NCCN criteria met*	Yes	49,218 (100%)	68,399 (84.5%)	117,617 (90.4%)	322,161 (86.7%)
	No	0	12,534 (15.5%)	12,534 (9.6%)	49,433 (13.3%)
Personal cancer	Ovarian	317 (0.6%)	1151 (1.4%)	1468 (1.1%)	–
	Pancreatic	56 (0.1%)	164 (0.2%)	220 (0.2%)	–
Family cancer history (1st or 2nd)**	Female Breast	22,216 (45.1%)	53,333 (65.9%)	75,549 (58.0%)	291,328 (78.4%)
	Male Breast	342 (0.7%)	1007 (1.2%)	1349 (1.0)	7809 (2.1%)
	Ovarian	4242 (8.6%)	12,212 (15.1%)	16,454 (12.6%)	141,909 (38.2%)
	Pancreatic	3143 (6.4%)	7375 (9.1%)	10,518 (8.1)	34,874 (9.4%)
	Prostate	6875 (14.0%)	14,027 (17.3%)	20,902 (16.1%)	45,243 (12.2%)
Self-reported ancestry	White/Non-Hispanic	24,696 (50.2%)	48,913 (60.4%)	73,609 (56.6%)	212,287 (57.1%)
	Black/African	5613 (11.4%)	7876 (9.7%)	13,489 (10.4%)	33,767 (9.1%)
	Hispanic/Latino	5355 (10.9%)	4792 (5.9%)	10,147 (7.8%)	32,359 (8.7%)
	Asian	2460 (5.0%)	2278 (2.8%)	4738 (3.6%)	7586 (2.0%)
	Ashkenazi Jewish	200 (0.4%)	477 (0.6%)	677 (0.5%)	2595 (0.7%)
	Native American	341 (0.7%)	571 (0.7%)	912 (0.7%)	3100 (0.8%)
	Middle Eastern	429 (0.9%)	453 (0.6%)	882 (0.7%)	2072 (0.6%)
	Pacific Islander	49 (0.1%)	67 (0.1%)	116 (0.1%)	317 (0.1%)
	Other	295 (0.6%)	364 (0.4%)	659 (0.5%)	1458 (0.4%)
	Multiple	2883 (5.9%)	3941 (4.9%)	6824 (5.2%)	24,278 (6.5%)
	None specified	6897 (14.0%)	11,201 (13.8%)	18,098 (13.9%)	51,775 (13.9%)

Dx, diagnosis

^*^NCCN version 3.2019

^**^All women included in this study were suspected of hereditary breast/ovarian cancer. Of these women, 4216 (0.8%) were missing family cancer history information and 43,094 (8.6%) had a family cancer history that was not accounted for by a first- or second-degree relative with female or male breast, ovarian, pancreatic, or prostate cancer

The proportion of breast cancers diagnosed in women with a family history of breast cancer is shown in Table 2**.** The absence of breast cancer in other family members was more common among affected women (42.0%) compared to unaffected women (21.6%). Among affected women, breast cancer in the family was more commonly reported at older ages (14.6% ≤ 45 versus 32.3% > 45). Conversely, the presence of breast cancer in the family was more commonly diagnosed at younger ages for unaffected women (39.1% ≤ 45 versus 29.5% > 45). This difference in age at diagnosis for breast cancer has been reported in other studies and is likely to be a function of how affected and unaffected women met criteria for testing, as a stronger family cancer history is typically required for unaffected women to meet NCCN testing criteria [4].

**Table 2:** Family history of female breast cancer among first- and second-degree relatives of affected and unaffected women

Family history of breast cancer	Affected with breast cancer	Unaffected
No familial female breast cancer	54,602 (42.0%)	80,266 (21.6%)
Dx age ≤ 45	18,948 (14.6%)	145,133 (39.1%)
Dx age > 45	42,014 (32.3%)	109,548 (29.5%)
Breast cancer with missing age	14,587 (11.2%)	36,647 (9.9%)

Dx, diagnosis

### PV prevalence

PVs were identified in 6.0% (N = 30,115) of all women tested (Table 3)**.** PVs were more common in affected women (9.3%) compared to unaffected women (4.9%). Among affected women, PVs in *ATM* (0.9%), *BRCA1* (2.1%), *BRCA2* (2.3%), *CHEK2* (1.4%), and *PALB2* (0.8%) were most common. This trend was similar for unaffected women.

**Table 3:** PV distribution

Gene	Affected with breast cancer	Unaffected	Total
Any PV	12,071 (9.3%)	18,044 (4.9%)	30,115 (6.0%)
*ATM*	1205 (0.9%)	2030 (0.5%)	3235 (0.6%)
*CHEK2*	1845 (1.4%)	3032 (0.8%)	4877 (1.0%)
*PALB2*	1076 (0.8%)	1203 (0.3%)	2279 (0.5%)
*BRCA1*	2782 (2.1%)	3095 (0.8%)	5877 (1.2%)
*BRCA2*	2960 (2.3%)	3788 (1.0%)	6748 (1.3%)
Other	2203 (1.7%)	4896 (1.3%)	7099 (1.4%)
None	118,080 (90.7%)	353,550 (95.1%)	471,630 (94.0%)

**Fig. 1:**
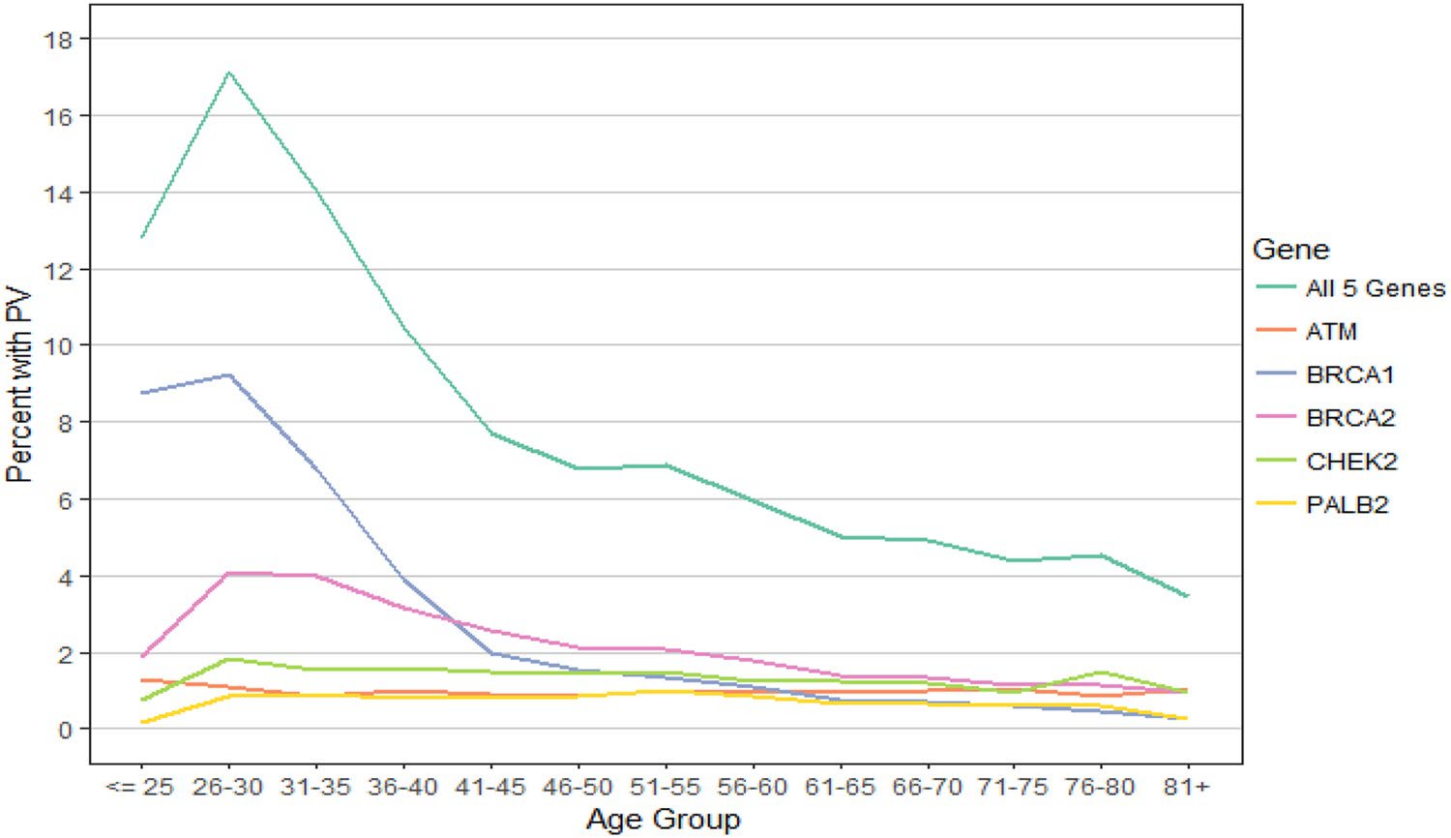
PV prevalence by age at diagnosis—affected women

The prevalence of PVs according to age at diagnosis for women with a personal history of breast cancer is shown in Fig. 1. When all PVs were considered, there was a peak in prevalence in the 26–30 age group, followed by a gradual decline with age. When PV prevalence was considered separately for each gene, the same decline with age was seen for *BRCA1* and, to a lesser extent, *BRCA2*. In contrast, PV prevalence in *ATM, CHEK2,* and *PALB2* was similar across all ages.

### Risk factors for carrying a PV

The risk of carrying a PV based on age at breast cancer diagnosis was evaluated for affected women (Table 4). After controlling for family history, women diagnosed with breast cancer ≤ 45 years of age were nearly four times more likely to carry a *BRCA1* PV than those diagnosed > 45 years (OR 3.95, 95% CI 3.64–4.29). Breast cancer diagnoses ≤ 45 years of age was associated with a more modest, but statistically significant, risk of carrying a PV in *BRCA2* (OR 1.98, 95% CI 1.84–2.14). This early age at diagnosis was associated with a small increased risk of carrying a PV in *CHEK2* (OR 1.34, 95% CI 1.21–1.47) or *ATM* (OR 1.22, 95% CI 1.08–1.37) and with a non-significant increased risk of carrying a PV in *PALB2* (OR 1.12, 95% CI 0.98–1.27).

**Table 4:** Associations of clinical factors with PV status among women with breast cancer

Clinical factor	Gene	Odds ratio	Lower 95% CI	Upper 95% CI	P-Value
Age of BC Dx (≤ 45 vs. > 45)	*ATM*	1.22	1.08	1.37	1.35E − 03
	*CHEK2*	1.34	1.21	1.47	4.34E − 09
	*PALB2*	1.12	0.98	1.27	9.40E − 02
	*BRCA1*	3.95	3.64	4.29	2.37E − 235
	*BRCA2*	1.98	1.84	2.14	4.54E − 70
	*ATM, CHEK2, PALB2*	1.24	1.16	1.33	1.14E − 10
	*BRCA1/2*	2.74	2.59	2.9	6.96E − 273
Female relative w/BC (Any vs. None)	*ATM*	1.47	1.30	1.66	1.02E − 09
	*CHEK2*	1.36	1.23	1.50	1.52E − 09
	*PALB2*	1.68	1.48	1.92	1.28E − 14
	*BRCA1*	1.91	1.76	2.07	1.87E − 54
	*BRCA2*	1.66	1.53	1.80	2.64E − 36
	*ATM, CHEK2, PALB2*	1.47	1.37	1.57	6.16E − 29
	*BRCA1/2*	1.78	1.68	1.88	4.39E − 85
Female relative w/BC (≤ 45 vs. > 45)	*ATM*	1.19	1.01	1.40	3.47E − 02
	*CHEK2*	1.21	1.06	1.39	4.83E − 03
	*PALB2*	1.24	1.05	1.46	1.33E − 02
	*BRCA1*	3.29	2.95	3.67	9.82E − 103
	*BRCA2*	1.90	1.71	2.10	1.73E − 34
	*ATM, CHEK2, PALB2*	1.21	1.11	1.33	2.20E − 05
	*BRCA1/2*	2.47	2.29	2.66	1.31E − 122

BC, breast cancer; CI, confidence interval; Dx, diagnosis

All models were adjusted for personal history of ovarian and pancreatic cancer, and family history of ovarian, pancreatic, prostate, male breast cancer, and ancestry

The risk of carrying a PV based on the presence or absence of a family history of female breast cancer was also examined. Affected women with familial breast cancer were 1.36- to 1.91-times more likely to carry a PV in a high- or moderate-penetrance breast cancer risk gene compared to those with no family history of breast cancer (Table 4). Unaffected women with familial breast cancer were about 2-times more likely to carry a PV in one of the high-penetrance breast cancer-risk genes (*BRCA1, BRCA2, PALB2*) compared to those with no familial breast cancer (Table 5). However, familial breast cancer was not as strong of a predictor of *ATM* or *CHEK2* PV status among unaffected women.

**Table 5:** Associations of clinical factors with PV status among women unaffected with cancer of any type

Clinical Factor	Gene	Odds Ratio	Lower 95% CI	Upper 95% CI	P-Value
Female relative w/BC (Any vs. None)	*ATM*	1.09	0.96	1.24	1.67E − 01
	*CHEK2*	1.24	1.11	1.38	8.00E − 05
	*PALB2*	2.12	1.74	2.58	7.16E − 14
	*BRCA1*	2.05	1.84	2.28	1.55E − 39
	*BRCA2*	1.81	1.65	2.00	1.87E − 33
	*ATM, CHEK2, PALB2*	1.29	1.20	1.40	1.52E − 11
	*BRCA1/2*	1.91	1.78	2.05	1.57E − 69
Female relative w/BC (≤ 45 vs. > 45)	*ATM*	0.95	0.86	1.06	3.88E − 01
	*CHEK2*	1.07	0.98	1.17	1.12E − 01
	*PALB2*	0.98	0.86	1.11	7.48E − 01
	*BRCA1*	2.34	2.13	2.56	6.64E − 72
	*BRCA2*	1.25	1.16	1.35	6.81E − 09
	*ATM, CHEK2, PALB2*	1.01	0.96	1.08	6.25E − 01
	*BRCA1/2*	1.64	1.54	1.73	1.25E − 60

BC, breast cancer; CI, confidence interval

All models were adjusted for family history of ovarian, pancreatic, prostate, male breast cancer, and ancestry

The risk of carrying a PV was also evaluated based on the earliest age at diagnosis among affected female relatives. Affected women with a familial breast cancer diagnosed ≤ 45 years of age were about 3-times more likely to carry a PV in *BRCA1* compared to those with familial breast cancer diagnosed > 45 years (OR 3.29, 95% CI 2.95, 3.67; Table 4). A more modest effect was observed for *BRCA2* (OR 1.90, 95% CI 1.71, 2.10), *PALB2* (OR 1.24, 95% CI 1.05, 1.46), *ATM* (OR 1.19, 95% CI 1.01, 1.40), and *CHEK2* (OR 1.21, 95% CI 1.06, 1.39). Similar trends were observed for unaffected women, where *BRCA1* (OR 2.34, 95% CI 2.13, 2.56) and *BRCA2* (OR 1.25, 95% CI 1.16, 1.35), were associated with a significant increase in risk among women with a relative diagnosed ≤ 45 versus > 45 years of age with breast cancer (Table 5).

Women < 40 years old have a greater relative risk of breast cancer attributable to a *BRCA1* mutation [26], whereas 65 years is commonly used as an upper age cutoff due to previous NCCN guidelines for genetic testing in breast cancer [19, 27, 28]. Therefore, to assess whether any age-associated differences in PV prevalence were driven by very young or very old ages at diagnosis, a sub-analysis of affected women diagnosed with breast cancer between the ages of 40 and 65 was performed (Supplemental Table 1). In this sub-group, age at breast cancer diagnosis was no longer a significant predictor of mutation status for *ATM* (OR 1.12, 95% CI 0.97–1.30) and *PALB2* remained non-significant (OR 1.05, 95% CI 0.90–1.23). There was no notable change to the overall contribution of family history of breast cancer and mutation status in this more limited age sub-group.

## Discussion

With multi-gene panel testing becoming a more common component of cancer prevention and treatment decisions, it is important to more effectively identify patients who are likely to carry a PV as candidates for testing. Historically, these criteria have been based on well-characterized phenotypes for *BRCA1/2* carriers. Here we examined the impact of two traditional markers of hereditary cancer risk (age at breast cancer diagnosis and family cancer history) on the risk of carrying a PV in the most commonly mutated breast cancer-risk genes: *ATM, BRCA1, BRCA2, CHEK2,* and *PALB2.*

Considered alone, PV prevalence was similar across a continuum of five-year age groups at diagnosis for the non-*BRCA* genes included in this analysis. When collapsing age at diagnosis into a dichotomous variable (≤ 45 and > 45 years) and controlling for family history in multivariable logistic regression models, there was a statistically significant, but very modest association between age at diagnosis and PV status for *ATM* and *CHEK2*, as well as stronger associations for *BRCA1* and *BRCA2*. The finding that age at breast cancer diagnosis alone may not be a useful predictor of PVs in some moderate- and high-risk genes is clinically relevant, as genetic testing decisions are often heavily weighted by this factor. Therefore, the clinical significance of using age at diagnosis for these genes must be scrutinized within the wider context of other risk modifiers.

The data presented here showed that the presence of familial breast cancer was more strongly associated with PV carrier status for *ATM, BRCA1*, *BRCA2*, *CHEK2*, and *PALB2*, in affected women (similar to previous studies) [29]. Breast cancer family history was not strongly associated with *CHEK2* or *ATM* PV status among unaffected women. For *ATM*, the PV rates of 0.9% in women affected with breast cancer and 0.5% in unaffected women were similar to those observed in the Women’s Health Initiative (0.73% in women diagnosed with breast cancer after menopause, 0.30% in unaffected women) and the CARRIERS study (0.78% in affected women and 0.41% in unaffected women) [18, 30]. Similarly, early age (≤ 45 years) at breast cancer diagnosis in affected women was not strongly associated with PV carrier status for the non-*BRCA* genes, particularly for the high-penetrant gene *PALB2*.

The present findings support previous work showing that PV prevalence is not solely dependent upon age of diagnosis for many genes. For example, a cohort of 10,000 women with breast cancer being evaluated by genetics providers found a prevalence of PVs in moderate- and high-risk genes of 5.6% among women 65 years and older [28]. Recently, Boddicker et al. reported in a population-based study of over 26,000 women over the age of 65, a PV frequency of 3.18% for women with breast cancer and 1.48% for unaffected women [19]. Similarly, a single-center study of sequential patients with breast cancer showed that the frequency of PVs in non*-BRCA* genes as a single group was independent of age at breast cancer diagnosis [5]. Case–control and observational studies have also shown that the prevalence of PVs in breast cancer-risk genes did not differ significantly by age among affected women who previously tested negative for *BRCA1/2* [7, 31, 32]. In addition, a nested case–control study using data from the Women’s Health Initiative found that 3.5% of post-menopausal women with breast cancer, unselected for family history, carried a PV in a breast cancer-related gene; no trend for age was seen in this sample [18]. However, an analysis of families with PVs in *PALB2* found that the breast cancer log(relative risk) estimate decreased with age from age 25 to age 75 years using a linear trend model [33]. This differs from the present study which showed that age at diagnosis did not have a significant impact on the presence or absence of PVs in *PALB2* and there was no significant difference in positivity rate by age.

While current testing criteria were originally developed based on the clinical features associated with PVs in *BRCA1/2*, these and other data demonstrate that these criteria are not necessarily appropriate for a broader range of other moderate- and high-penetrant risk genes. The accumulation of evidence will require adaptations of testing guidelines over time. Based on the findings herein this is particularly relevant for *PALB2* PV carriers, as guidelines currently recommend consideration of prophylactic mastectomy [4]. For women with breast cancer, a PV in *PALB2* could impact the extent of the surgery selected to treat their primary breast cancer and prevention of a secondary breast cancer.

Previous work has suggested that expanding the age at diagnosis testing criterion may be appropriate to improve the identification of PV carriers. Yadav et al. evaluated alternate age thresholds for selecting candidates for genetic testing and noted that increasing the age to 65 years would identify > 90% of PV-positive patients [27]; however, this age expansion would increase the number of women found to be PV-negative. The American Society of Breast Surgeons recommends that genetic testing for *BRCA1/2* and *PALB2* be made available to every women with a personal history of breast cancer [16]. There could be significant benefits to simplifying the genetic testing process among women with breast cancer, both in terms of increased access and higher testing uptake. The perceived negative consequences to this approach includes a higher VUS to PV ratio, especially in minority women [34], the inclusion of genes on some panels for which breast cancer risks are unclear and management strategies lacking, and a strain on already limited genetic testing resources [35]. However, these potential problems are being addressed by improving provider genomic literacy, as well as increased availability of genetics professionals through telemedicine and other alternative service models.

### Strengths and limitations of the study

One of the strengths of this study is the large sample size of women tested with a multi-gene panel that includes high-risk and moderate-risk genes. However, a limitation is that this is not a population-based study, but one of women who qualified for clinical genetic testing by current guidelines and thus was enriched for high-risk individuals. Through multivariable adjustment for clinical factors that are related to genetic testing, we produced effect estimates that may apply to the general population of breast cancer cases [25]. Statistical adjustments for personal history, type and age of family history, and ancestry using a multivariable logistic regression model further support previous findings that age at breast cancer diagnosis is not a strong predictor of carrying *ATM*, *CHEK2*, or *PALB2* PVs.

The current study data were collected from test requisition forms, which is a limitation. Notably, < 1% of patients were missing family history data, whereas self-reported ancestry data were unavailable for ~ 14% of the patients (Table 1). Although the test requisition forms used in the current study collected information on the number of unaffected sisters, daughters, and maternal and paternal aunts—which is highly informative when assessing familial risk—the lack of data on unaffected male relatives prevents an analysis of the strength of complete family cancer history versus family size [25]. Certain biases in family history reporting may also exist. A less complete family history may be collected for patients with cancer, as these patients tend to meet guidelines for genetic testing regardless of their family history [25]. There may also be racial disparities in family history reporting arising from hereditary risk assessment referrals, receipt of genetic testing, and other social determinants of healthcare between non-Hispanic White patients and other racial groups [3, 36, 37]. Differential family history reporting between cases and controls could have modest effects on penetrance estimates [25], which could also be affected by racial differences in family history reporting. Reassuringly, however, a recent study that compared patient report of family history to family history data collected on laboratory test requisition forms found a high concordance of 95% [36].

## Conclusions

Overall, these data contribute to the evidence that age at breast cancer diagnosis is not a strong predictor of carrying a PV across some moderate- and high-penetrance breast cancer genes. While early age at diagnosis was strongly associated with *BRCA1* and modestly with *BRCA2*, it was associated with only a very modestly (*ATM* and *CHEK2*) or no (*PALB2*) increased risk of carrying a PV in non-*BRCA1/2* breast cancer-associated genes. Risk modifiers such as the presence of a family history of breast and other cancers, tumor characteristics, single-nucleotide polymorphisms and breast density may hold more weight than age at diagnosis for some genes and may inform testing guidelines [35]. As more evidence regarding the breast cancer risk modifiers associated with PVs in these and other genes is generated, it is important that testing guidelines are continually optimized to ensure that all appropriate candidates are tested.

## Supplementary Information

Below is the link to the electronic supplementary material.

Supplementary file1 (DOCX 30 KB)

## Data Availability

The datasets generated during and/or analyzed during the current study are not publicly available due to patient confidentiality but are available from the corresponding author on reasonable request.
