# Supplementary material for: The association between age at breast cancer diagnosis and prevalence of pathogenic variants
Source: Breast Cancer Res Treat. 2023 Apr 21;199(3):617–26. doi: 10.1007/s10549-023-06946-8 (PMC10175307; doi:10.1007/s10549-023-06946-8)
Supplement: Supplementary file 1 — Supplementary file1 (DOCX 30 KB) [file 10549_2023_6946_MOESM1_ESM.docx]

*Supplemental Table 1. Associations of clinical factors with PV status among women aged 40-65 years with breast cancer*

| **Clinical Factor** | **Gene** | **Odds Ratio** | **Lower 95% CI** | **Upper 95% CI** |
| --- | --- | --- | --- | --- |
| Age of Breast Cancer Dx (≤45 vs. >45) | *ATM* | 1.12 | 0.97 | 1.30 |
|  | *CHEK2* | 1.23 | 1.10 | 1.38 |
|  | *PALB2* | 1.05 | 0.90 | 1.23 |
|  | *BRCA1* | 1.89 | 1.70 | 2.10 |
|  | *BRCA2* | 1.53 | 1.39 | 1.68 |
| Female Relative with Breast Cancer (Any vs. None) | *ATM* | 1.40 | 1.22 | 1.62 |
|  | *CHEK2* | 1.38 | 1.23 | 1.54 |
|  | *PALB2* | 1.72 | 1.48 | 2.00 |
|  | *BRCA1* | 1.69 | 1.52 | 1.89 |
|  | *BRCA2* | 1.57 | 1.43 | 1.72 |

**References**

1. Daly, M.B., et al. *NCCN Clinical Practice Guidelines in Oncology, Genetic/Familial High-Risk Assessment: Breast and Ovarian (Version 3.2019)*. NCCN Clinical Practice Guidelines in Oncology 2019 January 18, 2019 February 20, 2019]; Available from: <https://www.nccn.org/professionals/physician_gls/pdf/genetics_screening.pdf>.
